# Supplementary material for: Spatial regulation by multiple Gremlin1 enhancers provides digit development with cis-regulatory robustness and evolutionary plasticity
Source: Nat Commun. 2021 Sep 21;12:5557. doi: 10.1038/s41467-021-25810-1 (PMC8455560; doi:10.1038/s41467-021-25810-1)
Supplement: Supplementary file 1 — Supplementary Information [file 41467_2021_25810_MOESM1_ESM.pdf]

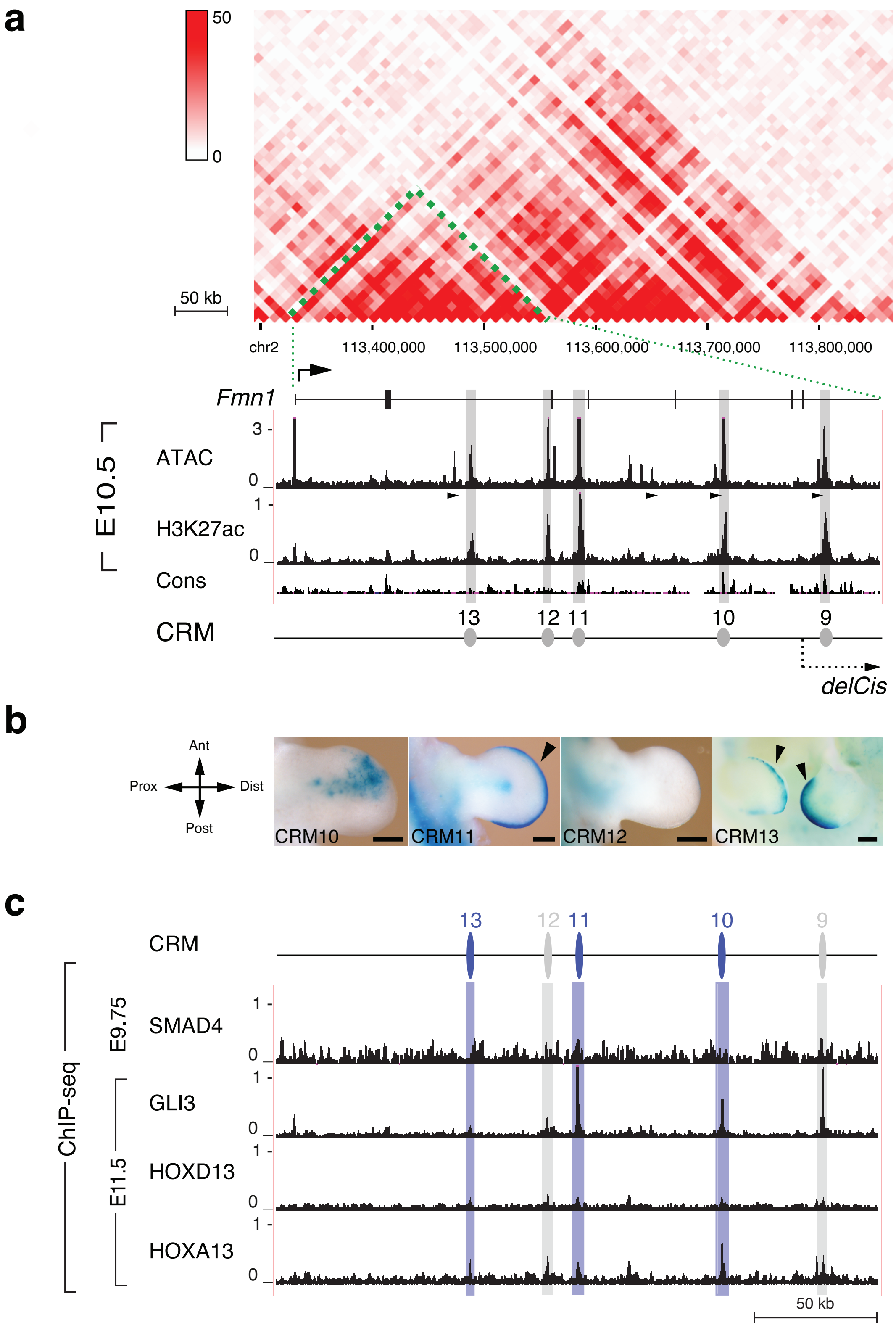

Supplementary Figure 1. Two of the CRMs in the *Fmn1* TAD are expressed in the AER.

a, Hi-C matrix of the *Grem1-Fmn1* TAD with the *Fmn1* TAD (~240kb) indicated by green

dashed lines. Colour intensity scale shows the contact frequencies. Shown below is an enlargement of the *Fmn1* TAD, with the direction of *Fmn1* transcription indicated by an arrow. ATAC-seq peaks (open chromatin; black arrowheads: CTCF sites) and H3K27ac ChIP peaks (active chromatin) identify four additional candidate CRMs in mouse forelimb buds (E10.5, CRM10-13). **b**, *LacZ* reporter assays in independent transgenic mouse founder embryos show that CRM11 (n=6/11) and CRM13 (n=12/16) are robust enhancers active in the AER (indicated by arrowheads), while CRM10 is expressed at low levels in the mesenchyme (n=2/4 expressors) and CRM12 has no reproducible enhancer activity (n=5) in forelimb buds at E11.5. The CRM11 and CRM13 activities in the AER are consistent with *Fmn1* being expressed by both the AER and mesenchyme, while *Grem1* is only expressed in the limb bud mesenchyme. The transgenic founder embryos that express *LacZ* in forelimb buds are indicated as the fraction of all embryos with *LacZ* expression in limb and non-limb tissues. Ant: anterior, Dist: distal, Post: posterior, Prox: proximal. Scale bar: 250 µm. **c**, ChIP-seq analysis detecting the interactions of SMAD4 (E9.75), GLI3 (E10.5) and HOXA13/D13 chromatin complexes in the *Fmn1* TAD during limb bud outgrowth (E11.5). There are no interactions of SMAD4 and HOXD13 with the CRMs detected in the *Fmn1* TAD, while GLI3 ChIP-seq peaks are detected in CRM10 and CRM11. In addition, a HOXA13 ChIP-seq peak is detected in CRM10. CRMs with enhancer activity are indicated in blue, CRMs with no activity in grey.

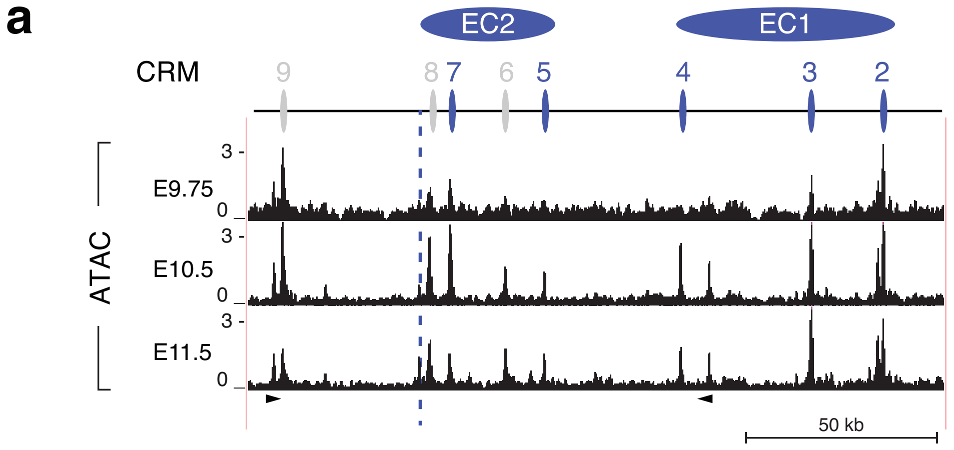

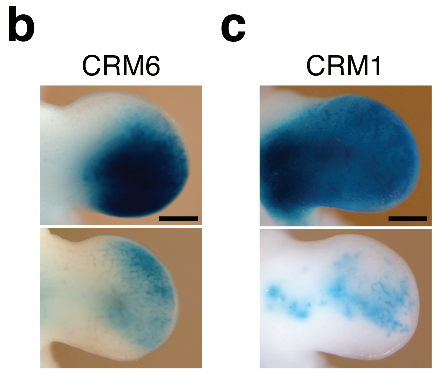

d

	SMAD4	GLI3	HOX13
CRM2	1	2	2
CRM3	0	1	1
CRM4	0	1	1
CRM5	0	1	1
CRM6	0	1	1
CRM7	0	1	1
CRM8	0	1	1

Supplementary Figure 2. Temporal progression of the CRM accessibilities in the *Grem1* TAD during mouse forelimb bud development. a, ATAC-seq analysis reveals the open chromatin profile during progression of forelimb bud development. During the onset of forelimb bud development at E9.75, CRM9 (located within the *Fmn1* TAD) and *CRM2* (located within the *Grem1* TAD (the TAD border is indicated as a vertical blue dashed line) appear highly accessible. In addition, the genomic regions of CRM3, CRM7 and CRM8 are also part of open chromatin regions, while no peaks for accessible chromatin are detectable for CRM4 to CRM6 in these early limb buds (E9.75). During forelimb bud outgrowth (E10.5 and E11.5), all CRMs in the *Grem1* TAD map to open chromatin regions. Note that the ATAC-seq profile shown for forelimb buds E10.5 is the same as in Fig. 1b. The CRM enhancers active in the mouse are indicated in blue and all other CRMs in grey. **b**, **c**, *LacZ* reporter assays in forelimb buds of independent transgenic mouse founder embryos show the variable activities of CRM6

(n=2/14 expressors) and CRM1 (n=5 strong, n=4 weak) at E11.0-11.5. The transgenic founder embryos that express *LacZ* in forelimb buds are indicated as the fraction of all embryos with *LacZ* expression in limb and non-limb tissues. Scale bars: 250 µm. **d**, Table listing the significantly enriched ChIP-seq peaks for the SMAD4, GLI3 and HOX13 transcriptional regulators in CRMs of the *Grem1* TAD.

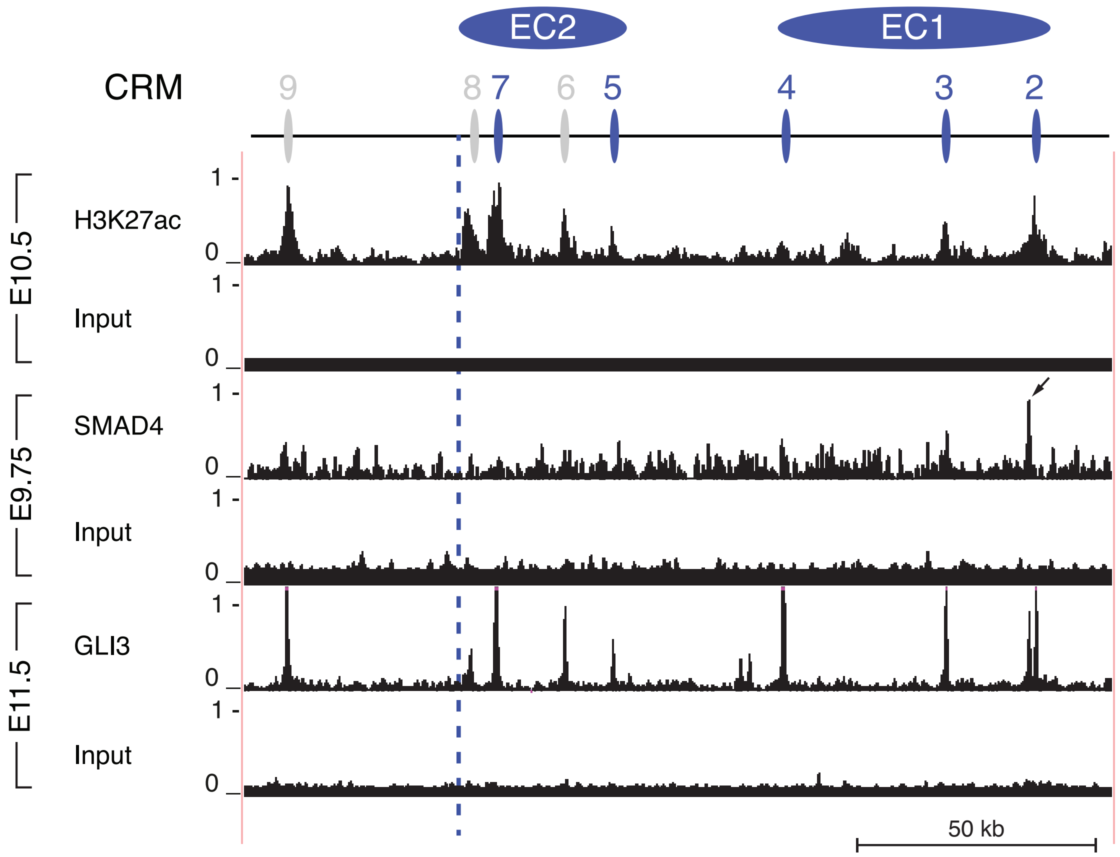

Supplementary Figure 3. ChIP-seq and input profiles for the novel mouse forelimb bud datasets generated as part of this study. ChIP-seq and input profiles at the same scale are shown for histone H3K27ac marks (E10.5), SMAD4^{3XF} (E9.75) and the GLI3^{3XF} transcription regulator (E11.5). The arrow indicates the only significantly enriched SMAD4 ChIP-seq peak in the *Grem1* landscape that is located in CRM2. The *Grem1* TAD border is shown as a vertical blue dashed line. The CRM enhancers active in the mouse are indicated in blue and all other CRMs in grey. The ChIP-seq profiles are the same as shown in Fig. 1b,d. For experimental details see the methods section.

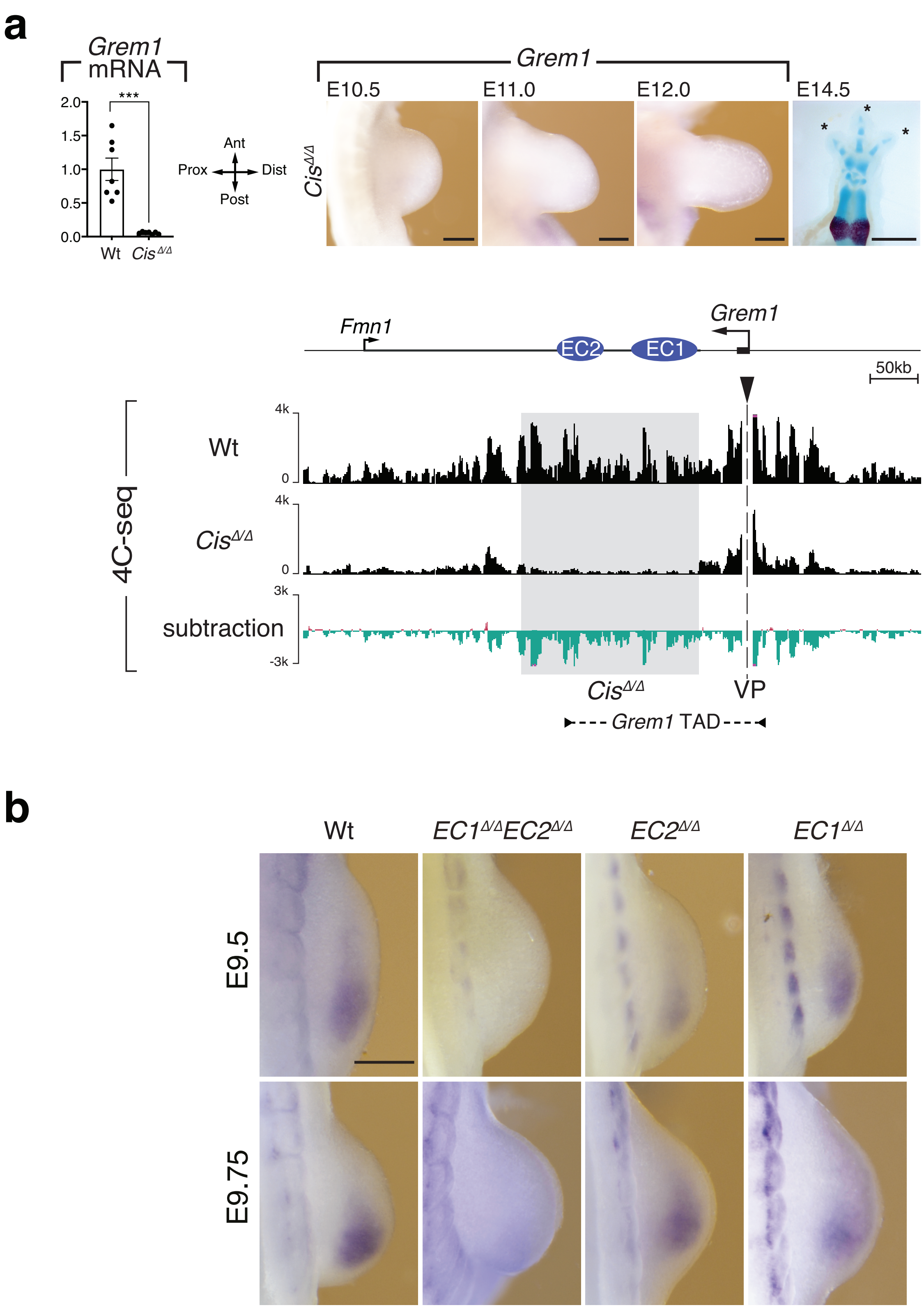

Supplementary Figure 4. Molecular analyses of the *delCis* (*Cis*$^{\Delta/\Delta}$) allele reveals wide-spread disruption of chromatin interactions. a, Analysis of *Cis*$^{\Delta/\Delta}$ forelimb buds. Upper panels: RT-qPCR analysis (n=7 independent biological replicates at ~E11.0, 40-42 somites).

Bars represent mean values +/- SEM. Exact *p*-value: ***p =0.000583; two tailed Mann-Whitney test. *Grem1* in situ hybridization shows the complete disruption of *Grem1* expression in $Cis^{\Delta/\Delta}$ forelimb buds (n=3 embryos analysed per stage from different litters and in minimally two independent experiments, scale bars: 250μm). Ant: anterior, Dist: distal, Post: posterior, Prox: proximal. A complete loss-of-function *Grem1* limb phenotype with digit reductions and associated loss of identity is observed in $Cis^{\Delta/\Delta}$ forelimb buds (asterisks, n=5, scale bar: 1mm). Lower panel: chromatin conformation capture (4C) using the *Grem1* promoter as viewpoint (VP, indicated by a black arrowhead) reveals the changes of chromatin interactions in $Cis^{\Delta/\Delta}$ forelimb buds (E11.0) in comparison to the wild-type controls. Subtraction after normalization reveals the differences in interactions with the proximal promoter in mutant forelimb buds (green: reduced and lost interactions). **b**, RNA in situ hybridisation shows the spatio-temporal *Grem1* distribution during the onset of limb bud development in wild-type (Wt), $EC1^{\Delta/\Delta}EC2^{\Delta/\Delta}$, $EC2^{\Delta/\Delta}$ and $EC1^{\Delta/\Delta}$ mutant forelimb buds (n= 3 embryos analysed per genotype and stage from different litters and in minimally two independent experiments). E9.5: 25-27 somites, E9.75: 28-29 somites. Scale bar: 200μm. Source data for the RT-qPCR analysis are provided as a Source Data file.

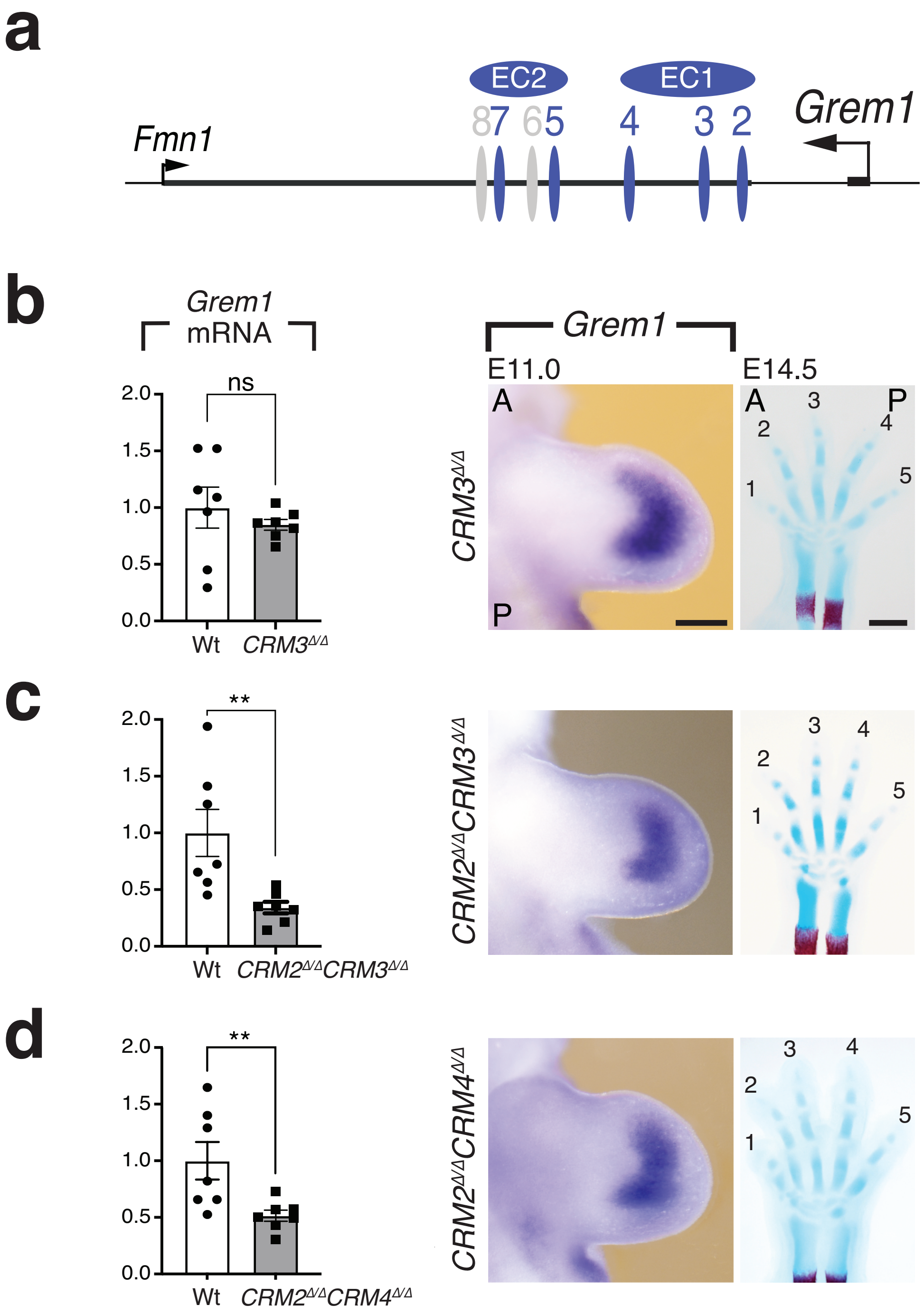

Supplementary Figure 5. *Grem1* expression and pentadactyly in compound mutants lacking two of the three CRMs in the EC1 cluster. a, Scheme of the *Grem1 cis*-regulatory landscape. The CRM enhancers active in the mouse are indicated in blue and all other CRMs in grey. **b-d,** Left panel: RT-qPCR analysis to determine the relative *Grem1* transcript levels in wild-type and homozygous forelimb buds (n=7 independent biological replicates at ~E11.0, 40-42 somites per genotype). Deletion of CRM3 does not cause a significant reduction in

Grem1 transcript levels (ns=not significant) Note: the ~50-60% reduced *Grem1* transcript levels in *CRM2*$^{\Delta/\Delta}$*CRM3*$^{\Delta/\Delta}$ (exact *p*-value: **p=0.002331) and *CRM2*$^{\Delta/\Delta}$*CRM4*$^{\Delta/\Delta}$ limb buds (exact *p*-value: ***p=0.009907) do not differ significantly from *CRM2*$^{\Delta/\Delta}$ and *EC1*$^{\Delta/\Delta}$ forelimb buds. Bars represent mean values +/- SEM. P-values were determined using the two tailed Mann-Whitney test. Middle panel: *Grem1* transcript distribution in mutant forelimb buds at E11.0. In *CRM2*$^{\Delta/\Delta}$*CRM3*$^{\Delta/\Delta}$ and *CRM2*$^{\Delta/\Delta}$*CRM4*$^{\Delta/\Delta}$ forelimb buds, the reduced *Grem1* domain is indistinguishable from *CRM2*-deficient forelimb buds (n=3 embryos analysed per genotype from different litters and in minimally two independent experiments). Scale bar: 250 µm. Right panels: limb skeletons at ~E14.5. Pentadactyly is maintained in all mutant forelimb buds (n≥3 embryos). Digits are shown from anterior (digit 1) to posterior (digit 5). Scale bar: 1mm. A: anterior, P: posterior. Source data for the RT-qPCR analysis are provided as a Source Data file.

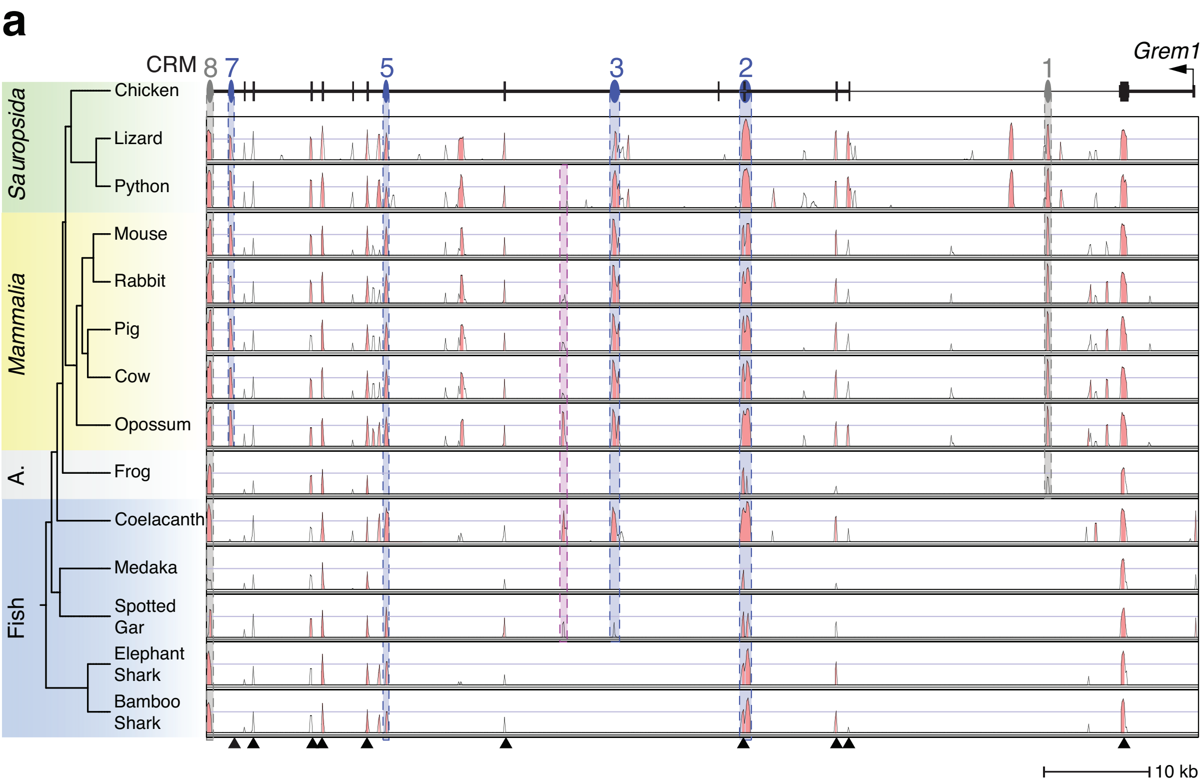

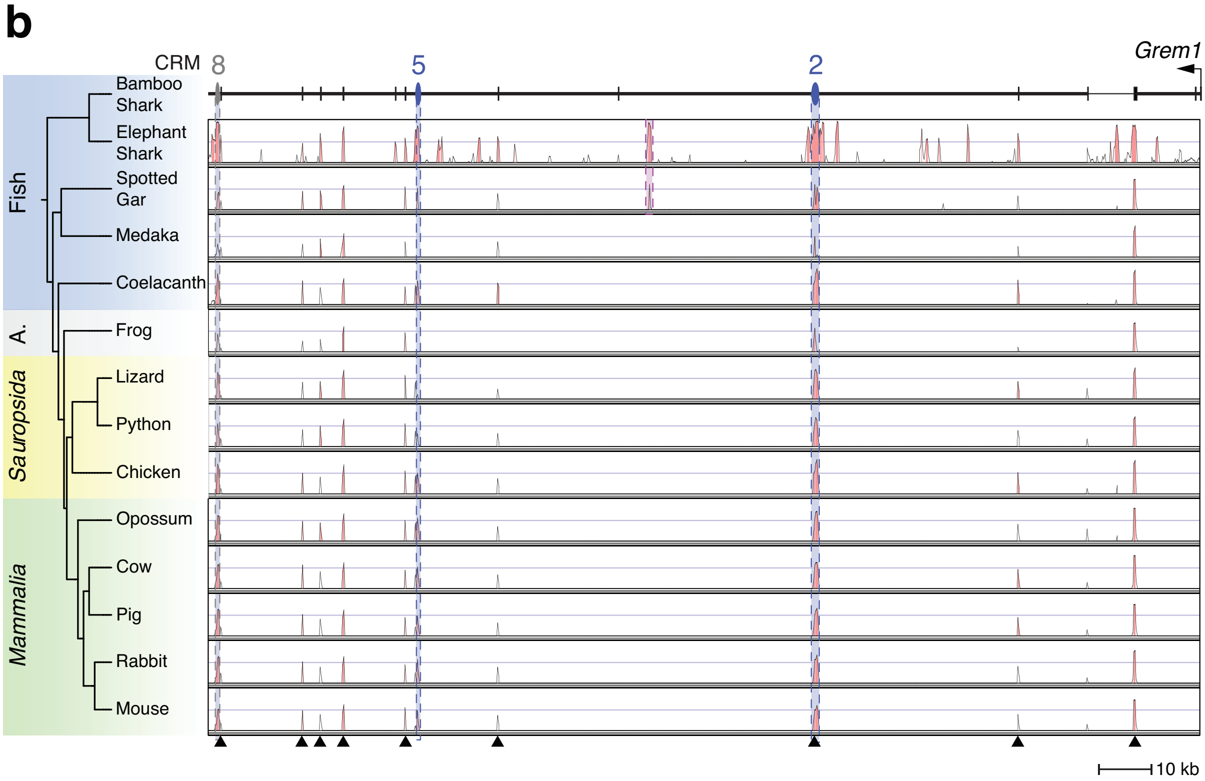

Supplementary Figure 6. Multiple sequence alignments using the chicken and bamboo shark genome as reference genomes. Alignment using the chicken (**a**) or bamboo shark (**b**) as a reference genome do not provide additional evidence for the presence of CRM4 or CRM6

in *Sauropsida* or basal fishes. In addition, there is no evidence for the presence of a CRM3 or CRM7-like element in cartilaginous fish. Only two additional conserved regions, likely not corresponding to additional *Fmn1* exons were identified using chicken (**a**) or bamboo shark (**b**) genome as a reference (highlighted in purple). Note that in both cases the regions are not deeply conserved nor do they overlap any of the functionally verified mouse CRM enhancers. The CRM enhancers active in the mouse are indicated in blue and all other CRMs in grey. Black arrowheads indicate the conserved *Fmn1* exons. A: amphibians.

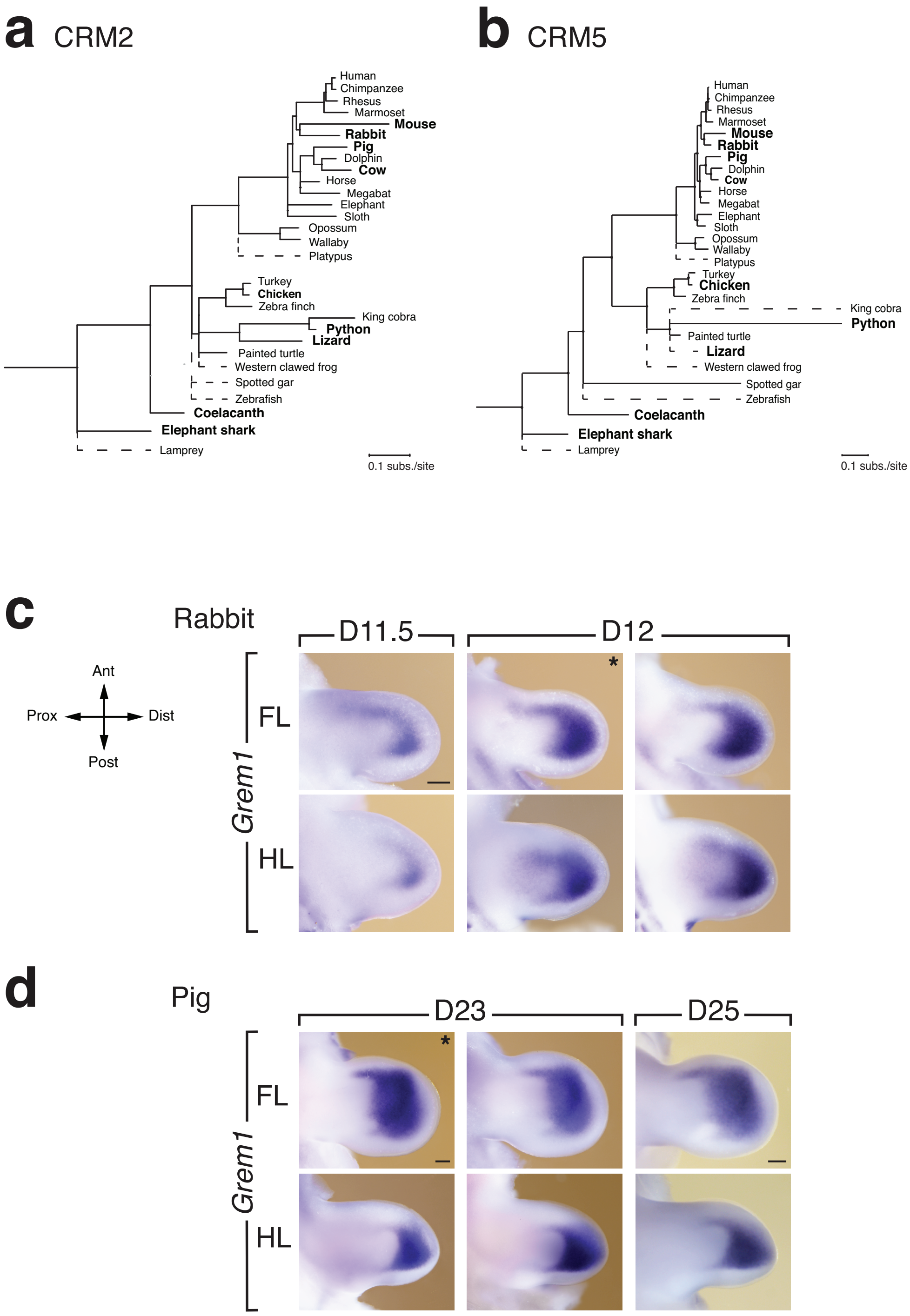

Supplementary Figure 7. Conservation and diversification of the CRM2 and CRM5 enhancers and spatial *Grem1* expression. a, b, Phylogenetic tree of 29 vertebrate species illustrates the evolutionary conservation CRM2 and CRM5 enhancers using the mouse genome (mm10) as reference. The branch length represents the bases substitution rate (scale bar corresponds to 0.1 substitutions per site). Dashed lines indicate the failure to detect the

enhancer in the particular species, possibly due to the stringent bidirectional best hits (BBH) approach used to finally obtain a robust phylogenetic tree. For the species indicated in bold, the CRM2 and CRM5 enhancer activities were assayed by *LacZ* reporters in transgenic mouse embryos. **c, d**, Spatial distribution of *Grem1* transcripts during limb bud outgrowth in rabbit (panel c, n=3 embryos analysed at two stages from two different litters and in minimally two independent experiments) and pig embryos (panel d, n=3 embryos analysed at two stages from different litters and in minimally two independent experiments). Both forelimb (FL) and hindlimb (HL) buds were analysed at stages orthologous to mouse limb development at ~E11.0-E11.5. Asterisks indicate the panels used in main Fig. 4c to illustrate the rabbit and pig forelimb bud expression patterns. Scale bars: 250µm. Ant: anterior, Dist: distal, Post: posterior, Prox: proximal.

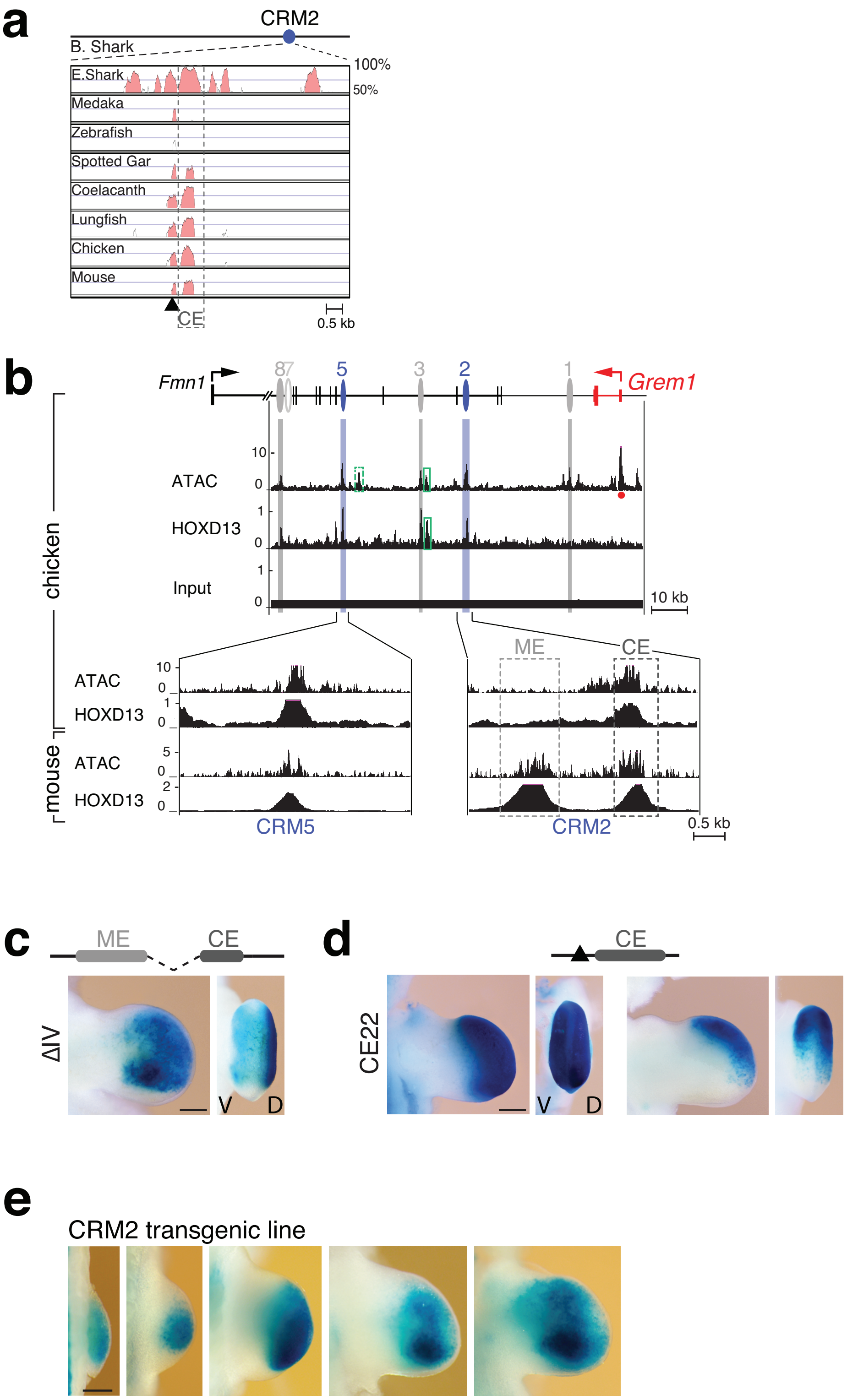

Supplementary Figure 8. The absence of a conserved ME region in chicken genome is supported by the lack of open chromatin and interaction with HOXD13 complexes in chicken wing buds. a, Conservation plot analysis for CRM2 using the bamboo shark genome as reference genome. **b,** The chicken *Grem1* TAD. Upper panels: ATAC-seq (wing buds, stage

HH24) and HOXD13 ChIP-seq analysis (wing buds stage HH27). The red dot indicates the *Grem1* transcription start site. This analysis reveals the absence of open chromatin and HOXD13 ChIP-seq peaks for the genomic locations corresponding to mouse CRM4 and 6 (not conserved in chicken) and for CRM7 (compare to Fig. 1b, d). In addition, a chicken-specific accessible chromatin region with a HOXD13 ChIP-seq peak (green solid box) and an ATAC-seq peak without a HOXD13 ChIP-seq peak (green dashed box) were detected. Lower panels: direct comparison of the chicken and the mouse ATAC-seq and HOXD13 profiles (E11.5) for the CRM2 and CRM5 regions (enlargements of the regions shown in Fig. 1b, d). The ATAC-seq and HOXD13 ChIP-seq profiles are very similar for the orthologous chicken and mouse CRM5 regions. In addition, the deeply conserved CE region of CRM2 maps to open chromatin and interacts with HOXD13 transcription complexes in both species (black dashed box). In contrast, the chicken genomic region upstream of CE lacks ATAC-seq and HOXD13 ChIP-seq peaks detected in the corresponding mouse ME region (grey dashed box). **c,** Deletion of the intervening genomic region (ΔIV) between ME and CE in CRM2 results in a *LacZ* activity pattern (n=3/4) identical to the MECE reporter construct (lower panel, Fig. 5c). **d**, A reporter construct encoding the deeply conserved region encompassing the CE region and *Fmn1* exon 22 (CE22) is active in the entire distal limb bud mesenchyme (n=2/3, left panels). In one case (n=1/3, right panel), the *LacZ* activity is similar to the one of the CE region alone (middle panel, Fig. 5c). Panels c,d: V = ventral; D = dorsal. The transgenic founder embryos that express *LacZ* in forelimb buds are indicated as the fraction of all embryos with *LacZ* expression in limb and non-limb tissues (panel c,d). **e,** Temporal analysis of the *LacZ* distribution during mouse limb bud development in a stable transgenic line expressing the CRM2 transgene (n=3 embryos analysed per stage from different litters and minimally two independent experiments). This analysis reveals that the CRM2 enhancer is active from the onset of limb bud development with a distinct posterior bias in its expression domain during progression of mouse limb bud

outgrowth. The spatial expression domain at later limb bud stages bears similarity with the *LacZ* activities of the MECE (Fig. 5c) and ΔIV transgenic constructs (panel **d**). This confirms that the non-coding ME and CE regions are essential elements of the CRM2 enhancer in mouse limb buds. Scale bars (panel c-e): 250 µm.

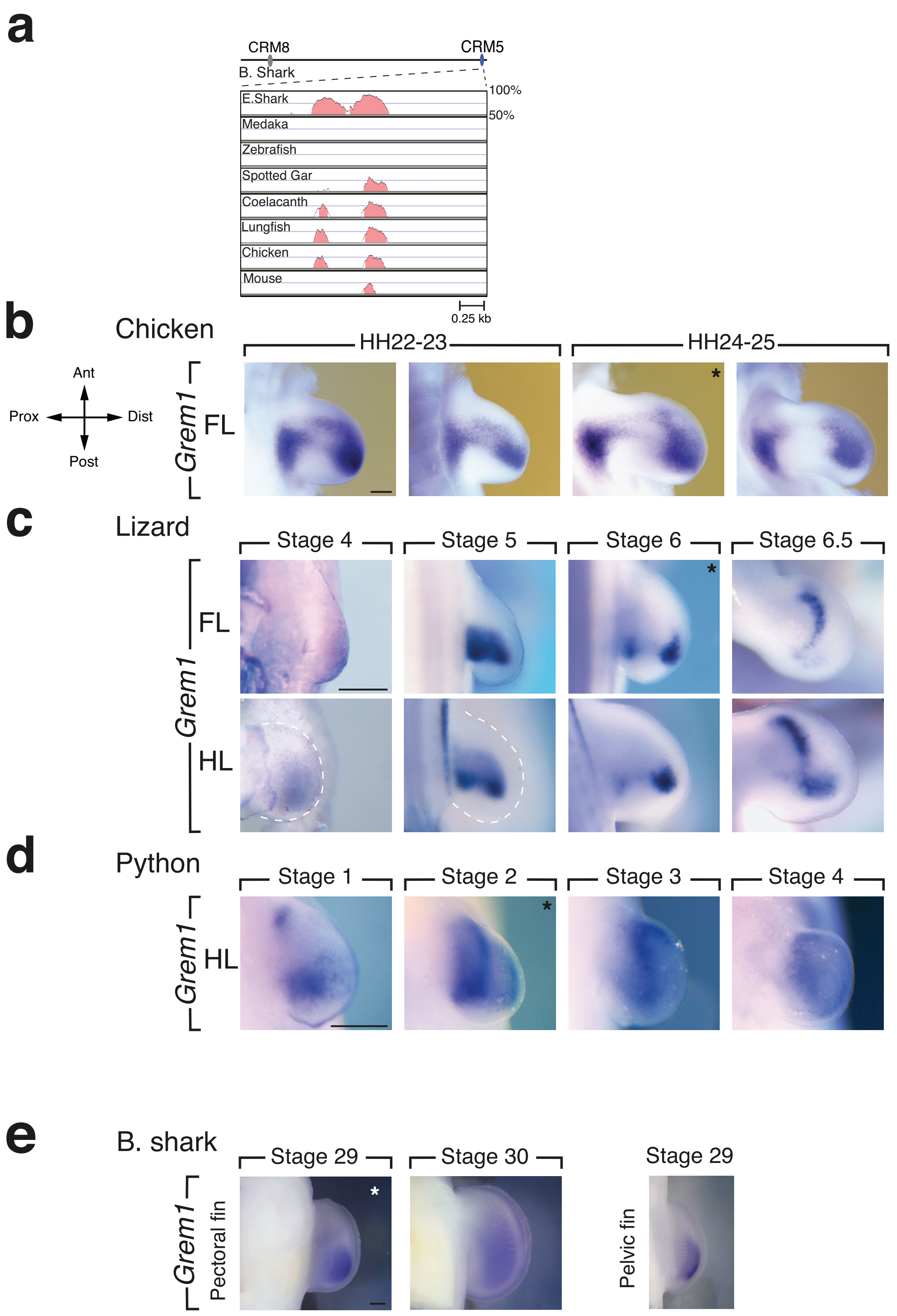

Supplementary Figure 9. *Grem1* expression in limb buds of *Sauropsid* species and bamboo shark fin buds. a, Conservation plot analysis for CRM5 using the bamboo shark genome as reference genome. **b**, Spatial distribution of *Grem1* in chicken wing buds (n=2). Patterns are

identical to the previously described *Grem1* expression dynamics in chicken limb buds)[69]. **c, d**, Spatial distribution of *Grem1* transcripts during outgrowth in lizard fore- and hindlimb buds (panel c, n=4 independent biological replicates) and in vestigial python hindlimb buds (panel d, stage 1: n=6; stage 2: n=4; stage 3: n=4; stage 4: n=2 independent biological replicates). Staging as described by Leal and Cohn, 2016[32]. **e,** Spatial distribution of *Grem1* transcripts in developing fin buds of brown-banded bamboo shark embryos (*Chiloscyllium punctatum*). *Grem1* expression in (paired) pectoral fin buds: at embryonic stage 29, *Grem1* expression is restricted to the posterior-distal mesenchyme (left panel, n=1), while at embryonic stage 30 the expression domain is enlarged (right panel, n=1). In addition, *Grem1* expression is also shown in a pelvic fin bud at embryonic stage 29 (n=1). Asterisks indicate the panels used for Fig. 6b, c. Ant: anterior, Dist: distal, Post: posterior, Prox: proximal. Scale bars (panel b-e): 250 µm.

Deletion	Coordinates (mm10)	sgRNA sequence	Size [kb]
ΔCRM2	chr2:113,689,603-113,699,473	AGCGGCAGTTCGGCTTCCGG	9.9
		TCTCATACGATCCAGGAGAA	
ΔCRM3	chr2:113,672,786-113,681,077	CACCGCCTGTGATCCATCGAATGCC	8.6
		AAACGGCATTCGATGGATCACAGGC	
ΔCRM4	chr2:113,640,518-113,641,588	CACCGCAGCCTATAATTCTCAGCGC	1.3
		AAACGCGCTGAGAATTATAGGCTGC	
ΔCRM5	chr2:113,603,760-113,606,572	CTTGTAATGCTAGGACGGCC	2.8
		AGTCAAGGACACACCTGTA	
ΔEC1	chr2:113,636,776-113,707,974	CACCGTGGCTTACCAGACTAGCGGT	71.2
		CACCGAATGGTCTGATCGCCA	
ΔEC2	chr2:113,572,310-113,606,574	TTCAGCTGCATTGCGTCCTA	34
		AGTCAAGGACACACGCTGTA	
delCis	chr2:113,528,475-113,709,592	described in reference 13	181

Supplementary Table 1. Genomic coordinates and guide RNA sequences to delete CRMs and enhancer clusters in the *Grem1* TAD for functional analysis.

Deletion	Forward	Reverse	Amplicon (bp)
CRM2 Δ	AACAAACAGTGCAATTC TGAAGAG	AGGAAGCCAGTGATCTC AAATATC	605
CRM2 Wt	GACTGGGATACATGTGA TGGTAAA	AGGAAGCCAGTGATCTC AAATATC	932
CRM3 Δ	CCTTTACATGCACACTC ACACA	TGCTTTGGATGTCTACAA GTGG	296
CRM3 Wt	AGCAGTGTGTCCCCCTA ATGAGC	GAGCCTGGAGCTTACTG AGCAAC	389
CRM4 Δ	AATTGAAGGAACAAAAG GCTCA	GGCTGACAGTAGTTTGC TGTTG	205
CRM4 Wt	GGCCTTCGAAACCATGA TGC	GGGAGGTGCTGGAATTA GGG	513
CRM5 Δ	CTCCCATATGCTCACCGGTTT	GGTGGGAGTGGAGTTTGACC	429
CRM5 Wt	GGTAAGGAGCCAGCCATATT TG	GGTGGGAGTGGAGTTTGACC	404
EC1 Δ	GGGACAAGTCACAGATCTTTT TG	TCCTTCATGTCTCGTTTTGTTT T	439
EC1 Wt	TCCAGTTAAATGCAAAAAGGA AA	CTCTTCCTTCATCTCTCCTAG CC	593
EC2 Δ	AGAAGCACTTGGCATGTGTG	TGAAAGAGTCGGCAACTGTG	509
EC2 Wt	AGAAGCACTTGGCATGTGTG	GGAAATTCAGAGCCATCCAA	712
dCis Δ	GGAGCGGATCTCAAACTCTC CTC	CTGCATTCTAGTTGTGGTTTG TCC	350
dCis Wt	GAAAGACTGCTGCAGAAGGA AGC	TCCAAAGAAGGCACAGGGGA CTT	200

Supplementary Table 2. Primer sequences used to genotype the *Grem1* alleles analyzed.

Name	Sequence
Grem1_C1_TUA	AATGATACGGCGACCACCGAGATCTACACTCTTTCCCTACACGACGCTCTTCCGATCTCTTCCGTAGATGCTGGCCGAT
Grem1_C6_index12	CAAGCAGAAGACGGCATACGAGAT**TACAAG**GTGACTGGAGTTCAGACGTGTGCTCTTCCGATCTAAGCACCAGGACCGAGTTTG
Grem1_C6_index2	CAAGCAGAAGACGGCATACGAGAT**ACATCG**GTGACTGGAGTTCAGACGTGTGCTCTTCCGATCTAAGCACCAGGACCGAGTTTG
Grem1_C6_index4	CAAGCAGAAGACGGCATACGAGAT**TGGTCA**GTGACTGGAGTTCAGACGTGTGCTCTTCCGATCTAAGCACCAGGACCGAGTTTG
Grem1_C6_index5	CAAGCAGAAGACGGCATACGAGAT**CACTGT**GTGACTGGAGTTCAGACGTGTGCTCTTCCGATCTAAGCACCAGGACCGAGTTTG
Grem1_C6_index6	CAAGCAGAAGACGGCATACGAGAT**ATTGGC**GTGACTGGAGTTCAGACGTGTGCTCTTCCGATCTAAGCACCAGGACCGAGTTTG
Grem1_C6_index7	CAAGCAGAAGACGGCATACGAGAT**GATCTG**GTGACTGGAGTTCAGACGTGTGCTCTTCCGATCTAAGCACCAGGACCGAGTTTG
Grem1_C6_index9	CAAGCAGAAGACGGCATACGAGAT**CTGATC**GTGACTGGAGTTCAGACGTGTGCTCTTCCGATCTAAGCACCAGGACCGAGTTTG

Supplementary Table 3. Universal and barcoded primers for preparation of 4C libraries. These primers were used for PCR amplification. The barcodes are highlighted in bold.

Mouse CRM	mm10 Coordinates	Size [bp]	Primer			
CRM1	chr2:113,724,491-113,724,715	225	fwd	GCATGTTTTTGGCCTTCATT	rev	TGAATCCCGCTATTGGTAGG
CRM2	chr2:113,690,950-113,694,587	3638	fwd	GGAATCCTGGCGATTAGTCA	rev	ACCTCCTGTAGCCTGGGATT
CRM3	chr2:113,674,074-113,675,273	1246	fwd	CCGCGTAGTTTCCATTCAAC	rev	GCAACTGCCTTGAAGGTGAT
CRM4	chr2:113,640,376-113,641,758	1389	fwd	TTCCTCAGAGCAGCTGAGTGT	rev	AGCGCTGAGAATTATAGGCTGA
CRM5	chr2:113,605,542-113,606,069	528	fwd	GAAGTCCCTCGAACACCAAG	rev	CTTTTCCAGCAAGGTGTGGT
CRM6	chr2:113,595,444-113,596,148	705	fwd	AGGTGTTGAGCCACTACTGC	rev	TCATCTTTTGTGAGGGGAAAA
CRM7	chr2:113,581,140-113,581,955	1029	fwd	GGTGCTCGAGAGCAGAAGTT	rev	ATACCAGGTGTGGTGGGTGT
CRM8	chr2:113,575,889-113,576,624	736	fwd	GGGATGAAAGAACGCCTGTA	rev	TGTCACTCTCCTGTCCTCCA
CRM9	chr2:113,537,701-113,538,435	735	fwd	TCATTCACCGTTGTCCATGT	rev	TGCATCTTCTGACGCTTGAC
CRM10	chr2:113,497,608-113,498,439	832	fwd	ATGGTGTGGAACAAGGCTTC	rev	TGCATCTTCTGACGCTTGAC
CRM11	chr2:113,439,964-113,441,240	1277	fwd	TCTTTCTCTGTCCCTTCC	rev	GCTCTCTCAGTTTATCCGC
CRM12	chr2:113,427,755-113,429,093	1339	fwd	TTTATGAGGGTCTGTCTCCTC	rev	AGGGACTGAGGCACAGAG
CRM13	chr2:113,397,272-113,398,270	999	fwd	AAGGAAATCTGTGGTTGGATAATG	rev	AAGGGCACATGATTGTACAG
CRM2 CE22	chr2:113,692,890-113,694,066	1177	fwd	CTCCTGTGTCGGGGAATAGA	rev	TCTCAGTGTTTTCGGCAGTG
CRM2 CE	chr2:113,693,196-113,693,953	758	fwd	TATCCAAGGCCCATGAAGTG	rev	GAGATAAGACCTGCTTACAGTTCCA
CRM2 ME	chr2:113691484-113692383	900	fwd	CATGGTGAACGATGCAAGAA	rev	ACAACAAAGAGACATTCTTGTGAACT
CRM2 MECE	See individual elements	1658	fwd 1.PCR	CATGGTGAACGATGCAAGAA	rev 1.PCR	ACAACAAAGAGACATTCTTG
			fwd 2.PCR	CAAGAATGTCTCTTTGTTGTTATCCAAGGCCCATGAG	rev 2.PCR	GAGATAAGACCTGCTTACAGTTCCA
CRM2 ΔME	chr2:113,690,950-113,694,587 deletion: chr2:113,691,577-113,692,277	2938	fwd 1.PCR	GGAATCCTGGCGATTAGTCA	rev 1.PCR	GGATAATGCTGAAGTCTATTCTTTCCCTACATCT
			fwd 2.PCR	AATAGACTTCAGCATTATCCAAGAGTCTACCTT	rev 2.PCR	ACCTCCTGTAGCCTGGGATT
CRM2 ΔCE	chr2:113,690,950-113,694,587 deletion: chr2:113,693,234-113,693,929	2942	fwd 1.PCR	GGAATCCTGGCGATTAGTCA	rev 1.PCR	TACAGTTCCAGACAACTGTGAGCCAGGC
			fwd 2.PCR	CACAGTTGTCTGGAACTGTAAGCAGGTC	rev 2.PCR	ACCTCCTGTAGCCTGGGATT
CRM2 ΔIV	chr2:113,690,950-113,694,587 deletion: chr2:113,692,277-113,693,233	2678	fwd 1.PCR	GGAATCCTGGCGATTAGTCA	rev 1.PCR	AGAAAAGAGCGTGACCTGCCCCGTGAAG
			fwd 2.PCR	GGCAGGTCACGCTCTTTTCTTTTGCATCAAGTCAAC	rev 2.PCR	ACCTCCTGTAGCCTGGGATT

Supplementary Table 4. Genomic coordinates and primers for amplification of the mouse candidate CRM regions. These CRM regions were cloned into the Hsp68-*LacZ* reporter vector and their limb bud enhancer potential assessed in transient transgenic mouse embryos. Also shown are the coordinates and primers for the mouse CRM2 CE and ΔCE constructs.

CRM Orthologues (Assembly)		Coordinates	Size [bp]	Primer
Rabbit (oryCun2)	CRM2	chr17:37,975,512-37,978,864	3353	GGCTAGAAAGCATGCCAGAG
				TAGTCCCTGAGGTCCCAAA
	CRM5	chr17:38,073,904-38,074,665	762	CCCAGAGCTCCTCACTTCAG
				TCTCAAAGCTCTCATCTTGCTG
Pig (susScr11)	CRM2	chr1:136,729,218-136,732,525	3308	GTCCCTTCTCTCTTCCATGC
				AATGGTGGAAAGATGTGTGG
	CRM5	chr1:136,845,296-136,846,106	811	GTGCCCTGGGGTAGACTGTA
				GCGGTTGGATGTTCTCATCT
Cow (bosTau9)	CRM2	chr10:29,858,417-29,861,217	2801	CCCAAAATCCCTTCCAGTTT
				CTGTGTGTGTGTGTCTGTATGTG
	CRM5	chr10:29,740,419-29,741,218	800	GCAGGAAAGCTGAACAATACCT
				GGCTTAAAGAGATGCTCTATGG
Chicken (galGal5)	CRM2	chr5:30,543,750-30,546,269	2520	GAGTCACTCCTGGCTCAGAAA
				ATTCCAGCGGGAAAACACT
	CRM5	chr5:30,511,368-30,512,133	766	TTGTTGCATTGAGGTTATTTTCA
				GCTATTTCAGACACAGTAGGTGACA
Python (molurus bivittatus-5.0.2)	CRM2	NW_006535812.1 [79018-80265]	1249	AGAGATTAATTAGGGGAACGTACAA
				TTGGACTGGGAAACGCTTA T
	CRM5	NW_006535812.1 [58984-59576]	592	CCAGGGCAGAGTGTATCGTC
				TTTTGATCATTCTGAAATACTGGAA
Lizard (anoCar2)	CRM2	chr1:32,920,998-32,922,337	1340	Synthesized at IDT
	CRM5	chr1:32,943,808-32,944,258	451	
Coelacanth (latCha1)	CRM2	JH126706:163,091-163,988	898	Synthesized at IDT
	CRM5	JH126706:85,817-86,468	652	
Elephant Shark (calMil1)	CRM2	KI635863:7,047,935-7,048,988	1054	AAGCACAGGGAAGTTGCAGT
				GTGCAAAGGGTGGGATCTTA
	CRM5	KI635863:7,083,856-7,084,336	481	CAAAACGCTTTGGCTTGTAG
				CACGCAAACTACCCATAGCA
Bambo Shark (Cpunctatum v1.0)	CRM2	BEZZ01000101:155,083-156,296	1214	Synthesized at IDT
	CRM5	BEZZ01000101:230,775-231,228	454	

Supplementary Table 5. Genomic coordinates and primers for amplification of the CRM2 and CRM5 regions from different species.

Gene	Forward	Reverse	Size [bp]
Grem1	CCCACGGAAGTGACAGAATGA	AAGCAACGCTCCCACAGTGTA	53
Rpl19	ACCCTGGCCCGACGG	TACCCTTTCCTCTTCCCTATGCC	53

Supplementary Table 6. RT-qPCR primers to amplify specific region of the *Grem1* and *RPL19* transcripts.

Common Name	Scientific Name	Source and Assembly	Coordinates of the *Grem1-Fmn1* TAD	Strand
Mouse	Mus musculus	UCSC mm10	chr2:113,313,824-113,779,181	+
Rabbit	Oryctolagus cuniculus	UCSC oryCun2	chr17:37,872,491-38,409,484	-
Pig	Sus scrofa	UCSC SusScr11	chr1:136,603,077-137,170,757	-
Cow	Bos Taurus	UCSC bosTau9	chr10:29442507-30045950	+
Opposum	Monodelphis domestica	UCSC monDom5	chr1:188,412,539-189,022,183	+
Chicken	Gallus gallus	UCSC galGal5	chr5:30,362,697-30,645,411	+
Zebra finch	Taeniopygia guttata)	UCSC taeGut2	chr5:29,874,60- 30,066,547	+
Emu	Dromaius novaehollandiae	NCBI droNov1 (GCF_003342905_1)	NW_020451784.1:146713-335467	+
Python	Python bivittatus	NCBI Python molurus bivittatus-5.0.2	NW_006535812.1[1-124418]	+
Lizard	Anolis carolinensis	UCSC AnoCar2.0	chr1:32,873,848-33,105,463	-
Tibetan Frog	Nanorana parkeri	UCSC nanPar1	KN905994v1:660,625-877,435	-
Lungfish	Neoceratodus forsteri	Genebank JADMNL010000017.1	JADMNL010000017.1:20676886-24096076	-
Coelacanth	Latimeria chalumnae	UCSC LatCha1	JH126706:1-214,337	+
Spotted Gar	Lepisosteus oculatus	NCBI LepOcu1	ChrLG7:15,355,746-15,528,614	+
Zebrafish	Danio rerio	UCSC danRer11	chr20:29,434,817- 29,471,775	+
Japanese Medaka	Oryzias latipes	NCBI ASM223467v1	Chr22:14,311,286-14,367,948	+
Elephant Shark	Callorhinchus milii	UCSC calMil1	KI635863:7,007,558-7,161,090	-
Bamboo Shark	Chiloscyllium punctatum	NCBI Cpunctatum_v1.0	BEZZ01000101:76,696-456,393	-

Supplementary Table 7. Species names, genome assemblies and coordinates of the *Grem1-Fmn1* TADs used for VISTA conservation plot analysis.

Common Name	Species	Assembly
Human	*Homo sapiens*	hg19
Chimpanzee	*Pan troglodytes*	panTro5
Rhesus	*Macaca mulatta*	rheMac8
Marmoset	*Callithrix jacchus*	calJac3
Mouse	*Mus musculus*	mm10
Rabbit	*Oryctolagus cuniculus*	oryCun2
Pig	*Sus scrofa*	susScr11
Dolphin	*Tursiops truncatus*	turTru2
Cow	*Bos taurus*	bosTau8
Horse	*Equus caballus*	equCab2
Megabat	*Pteropus vampyrus*	pteVam1
Elephant	*Loxodonta africana*	loxAfr3
Sloth	*Choloepus hoffmanni*	choHof1
Opossum	*Monodelphis domestica*	monDom5
Wallaby	*Macropus eugenii*	macEug2
Platypus	*Ornithorhynchus anatinus*	ornAna2
Turkey	*Meleagris gallopavo*	melGal5
Chicken	*Gallus gallus*	galGal5
Zebra finch	*Taeniopygia guttata*	taeGut2
King cobra	*Ophiophagus hannah*	ophHan1
Python	*Python molurus bivittatus*	molBiv5
Painted turtle	*Chrysemys picta*	chrPic1
Lizard	*Anolis carolinensis*	anoCar2
Western clawed frog	*Xenopus tropicalis*	xenTro9
Spotted gar	*Lepisosteus oculatus*	lepOcu1
Zebrafish	*Danio rerio*	danRer11
Coelacanth	*Latimeria chalumnae*	latCha1
Elephant shark	*Callorhinchus milii*	calMil1
Lamprey	*Petromyzon marinus*	petMar2

Supplementary Table 8. Species names and genome assemblies used for the phylogenetic tree inference analysis of the CRM2 and CRM5 enhancers.
