# Supplementary material for: Spatial regulation by multiple Gremlin1 enhancers provides digit development with cis-regulatory robustness and evolutionary plasticity
Source: Nat Commun. 2021 Sep 21;12:5557. doi: 10.1038/s41467-021-25810-1 (PMC8455560; doi:10.1038/s41467-021-25810-1)
Supplement: Supplementary file 3 — Reporting Summary [file 41467_2021_25810_MOESM3_ESM.pdf]

nature research

Corresponding author(s): Aimée Zuniga and Rolf Zeller

Last updated by author(s): Aug 13, 2021

Reporting Summary

Nature Research wishes to improve the reproducibility of the work that we publish. This form provides structure for consistency and transparency in reporting. For further information on Nature Research policies, see our Editorial Policies and the Editorial Policy Checklist.

Statistics

For all statistical analyses, confirm that the following items are present in the figure legend, table legend, main text, or Methods section.

n/a	Confirmed	
☐	☒	The exact sample size (*n*) for each experimental group/condition, given as a discrete number and unit of measurement
☐	☒	A statement on whether measurements were taken from distinct samples or whether the same sample was measured repeatedly
☐	☒	The statistical test(s) used AND whether they are one- or two-sided<br>*Only common tests should be described solely by name; describe more complex techniques in the Methods section.*
☒	☐	A description of all covariates tested
☐	☒	A description of any assumptions or corrections, such as tests of normality and adjustment for multiple comparisons
☐	☒	A full description of the statistical parameters including central tendency (e.g. means) or other basic estimates (e.g. regression coefficient) AND variation (e.g. standard deviation) or associated estimates of uncertainty (e.g. confidence intervals)
☐	☒	For null hypothesis testing, the test statistic (e.g. *F*, *t*, *r*) with confidence intervals, effect sizes, degrees of freedom and *P* value noted<br>*Give P values as exact values whenever suitable.*
☒	☐	For Bayesian analysis, information on the choice of priors and Markov chain Monte Carlo settings
☒	☐	For hierarchical and complex designs, identification of the appropriate level for tests and full reporting of outcomes
☒	☐	Estimates of effect sizes (e.g. Cohen's *d*, Pearson's *r*), indicating how they were calculated

Our web collection on statistics for biologists contains articles on many of the points above.

Software and code

Policy information about availability of computer code

Data collection	No software was used for data collection
Data analysis	Statistical analysis: PRISM 8 and 9 (software by GraphPad)<br>Primer design: Primer 3 (Web-based Interface, http://bioinfo.ut.ee/primer3-0.4.0/ Untergasser et al. 2012 doi: 10.1093/nar/gks596)<br>sgRNA design: CRISPOR v.4.99 (Web-based Interface http://crispor.tefor.net/crispor Haussler at al. 2016 10.1186/s13059-016-1012-2<br>Multiple sequence alignments & conservation plot analysis: VISTA (Web-based interface: http://genome.lbl.gov/vista/index.shtml; Frazer et al. 2004 10.1093/nar/gkh458; Jalview v1.8.3 and IQ-TREE v1.6.9 were used for the visualization of the multiple sequence alignment.<br>Phylogenetic trees were generated with the web tool phyloT v2 (https://phylot.biobyte.de/) and visualized with iTOL v6 (https://itol.embl.de/)<br>NGS analysis: The use of all software is described in the materials and methods section: Bowtie v2.2.9, MACS2 v2.1.1, SAMtools v1.7, Burrows-Wheeler Alignment (BWA) for Gli3 ChIP-seq, FastQC v0.11.4, BEDTools v2.26.0, R v3.5.0, Trim_Galore v0.4.1, QuasR v3.4.2 R package (Bioconductor v3.6). The repository containing custom scripts for generation of 4C-seq profiles is available at https://doi.org/10.5281/zenodo.5181231<br>TF binding site predictions:<br>Web based PWMscan tool (https://ccg.epfl.ch/pwmtools/pwmscan.php#) Ambrosini et al. 2018 10.1093/bioinformatics/bty127<br>Web based TrimAi v1.2 tool (http://phylemon2.bioinfo.cipf.es/) Salvador Capella-Gutierrez, 2009 10.1093/bioinformatics/btp348

For manuscripts utilizing custom algorithms or software that are central to the research but not yet described in published literature, software must be made available to editors and reviewers. We strongly encourage code deposition in a community repository (e.g. GitHub). See the Nature Research guidelines for submitting code & software for further information.

Data

Policy information about availability of data

All manuscripts must include a data availability statement. This statement should provide the following information, where applicable:

- Accession codes, unique identifiers, or web links for publicly available datasets
- A list of figures that have associated raw data
- A description of any restrictions on data availability

The newly genome-wide dataset generated for this study have been deposited in the gene expression omnibus (GEO) database. The ATAC-seq and H3K27ac datasets are available under the GEO accession number GSE151488 https://www.ncbi.nlm.nih.gov/geo/query/acc.cgi?acc=GSE151488 and the SMAD4 and GLI3 ChIP-seq and 4C datasets under the GEO accession number GSE151647 https://www.ncbi.nlm.nih.gov/geo/query/acc.cgi?acc=GSE151647.
Anolis sagrei Grem1 mRNA (partial) and Python regius Grem1 mRNA (partial) are available under: GenBank accession number MT124663 https://www.ncbi.nlm.nih.gov/nuccore/MT124663 and GenBank accession number KX778825 https://www.ncbi.nlm.nih.gov/nuccore/KX778825 respectively. No restrictions apply to data availability.

Field-specific reporting

Please select the one below that is the best fit for your research. If you are not sure, read the appropriate sections before making your selection.

- [x] Life sciences
- [ ] Behavioural & social sciences
- [ ] Ecological, evolutionary & environmental sciences

For a reference copy of the document with all sections, see nature.com/documents/nr-reporting-summary-flat.pdf

Life sciences study design

All studies must disclose on these points even when the disclosure is negative.

Sample size	For qualitative whole-mount in situ (WISH) analysis in mouse embryos n≥3 samples were analyzed per genotype and developmental stage (the exact numbers are included in the Figure legends). Gene expression patterns in embryos are extremely robust and based on the standard in the field and our previous experience (Benazet et al. 2009 10.1126/science.1168755) little to no technical and experimental variability is observed for embryos of the same stage and genotype. As we also use embryos from non-model organisms for analyses, in a few cases less than 3 embryos per stage were analyzed. For bamboo shark n=2 embryos were analyzed, but the spatial distributions in their paired fin buds are identical.<br>LacZ reporter assays: the standard in the field to study tissue-specific and spatially restricted enhancer activity is to analyse minimally three independent transgenic founder embryos that express the LacZ reporter in the tissue of interest. Enhancer unable to drive LacZ expression in the tissue of interest (limb buds), it has been suggested to analyze n≥5 independent transgenic founders showing at least some embryonic LacZ expression (Visel A, et al. 2007 10.1093/nar/gkl822). The sample size of this study are often even large with respect to the total number of LacZ expressing embryos.<br>Skeletal analysis: for scoring any limb skeletal/digit phenotypes caused by altered Grem1 expression that are 100% penetrant (=robust), analysing n≥ 3 embryos is standard. The is no clear guidelines for scoring variable (=non-robust) phenotypes, but samples sizes of n≥10 enables calculation of phenotypic penetrance frequencies (see Fig. 2e).<br>RT-qPCR assays: based on power calculations we have previously shown that a sample size of n=7 independent biological replicates per genotype is able to detect differences of 15-20% or more (Probst et al. 2009 10.1016/j.diff.2013.03.002)<br>ATAC-seq: For each developmental stage, two independent biological replicates (n=2) are used, which allow to determine that the replicates are consistent with one another. This is standard in the field; see https://informatics.fas.harvard.edu/atac-seq-guidelines.html).<br>4C-seq and ChIP-seq analysis: for each experiment, two independent biological replicates (n=2) were analysed to establish reproducibility, which is standard in the field (see e.g. the ENCODE guidelines https://www.encodeproject.org/about/experiment-guidelines/)
Data exclusions	None
Replication	Mouse WISH and skeletal analyses: n≥3 samples per genotype and stage isolated from different females were analyzed in minimally two independent experiments embryos. Also all relevant WISH were done by more than one experimenter in the group. All finding are highly reproducible, but also includes genotypes and stages with biologically relevant variability (see e.g. Fig. 2e). This allows us to conclude that variability observed in specific genetic make-ups is a consequence of disrupting biological robustness and not of technical nature.<br>LacZ reporter assays: each transgenic founder embryo is de facto an independent experiment as the random insertion into the genome is unique. Variation can be observed due to position effects of transgene insertion, therefore the activity of a limb bud enhancer was considered robust when similar or identical spatial activities were reproduced in n≥3 founder embryos. An enhancer was considered not active or non-robust when either no, low or highly variable or spurious activity was detected in transgenic limb buds. The fraction of the embryos with limb expression is indicated as n=x/y with x indicating the number of embryos with limb bud LacZ activity and y indicating the total number of embryos with LacZ activity (including limb bud and all other tissues).<br>RT-qPCR analysis: Each sample is a biological replicate and per sample three technical replicates are averaged to exclude experimental errors. This approach and using n=7 independent biological limb bud samples per genotype and from wild-type embryos in the same genetic background produces highly significant results. The seven samples per genotype were collected from different females at different days to avoid bias.<br>ATAC-seq: To assess the quality of biological replicates, the bamCount function of the R/Bioconductor package bamsignals (R version 3.4.2, Bioconductor version 3.6) was used to calculate a log-CPM (counts per million) normalized signal in bins of 500 bp across the genome. This signal between samples was compared using Pearson correlation coefficient and gave a good agreement between biological replicates (cor. coeff > 0.85). Two independent ATAC-seq datasets were used to establish the reproducibility (see previous section)

	4C-seq: two independent experiments were performed using limb buds from the different genotype together with wild-type controls from the same genetic background.<br>ChIP-seq: To assess the quality of biological replicates the bamCount function of the R/Bioconductor package bamsignals (R version 3.4.2, Bioconductor version 3.6) was used to calculate a log-CPM (counts per million) normalized signal in bins of 500 bp across the genome. Signal enrichment (= localization strength) of IP relative to the input was assessed by visualizing the cumulative signal density versus the cumulative genome size coverage. Both biological replicates agreed in the enrichment strength. Two or more experiments were performed for each ChIP-seq analysis using independently collected samples, which established reproducibility results.
Randomization	Randomisation is not possible, as due to the genetic complexity of the analysis, mouse embryos have to be genotyped prior to analysis. However, both male and females embryos were used and embryos from minimally 2 or more generations analyzed to avoid bias due to difference in sex and genetic background. All mice were kept in an outbred background (Swiss Albino) to avoid bias due to genetic drift.<br>For the comparative evolutionary analysis of Grem1 expression in different species limb bud of orthologous stages needed to be analyzed, which also precludes randomization
Blinding	The lacZ reporter activities was done blinded as founder embryos were stained and expression patterns scored prior to determining the genotypes (transgenic versus non-transgenic embryos).<br>For all experiments involving the comparative analysis of developmentally age-matched limb buds from genetically altered mouse embryos with often complex genotypes blinding was not possible. The same applies to comparative analysis of limb buds from different species<br>For genome-wide analysis, such as 4C, ChIP-seq and ATAC-seq blinding is not required (e.g. ENCODE guidelines https://www.encodeproject.org/about/experiment-guidelines/)

Reporting for specific materials, systems and methods

We require information from authors about some types of materials, experimental systems and methods used in many studies. Here, indicate whether each material, system or method listed is relevant to your study. If you are not sure if a list item applies to your research, read the appropriate section before selecting a response.

Materials & experimental systems

n/a	Involved in the study
☐	☒ Antibodies
☐	☒ Eukaryotic cell lines
☒	☐ Palaeontology and archaeology
☐	☒ Animals and other organisms
☒	☐ Human research participants
☒	☐ Clinical data
☒	☐ Dual use research of concern

Methods

n/a	Involved in the study
☐	☒ ChIP-seq
☒	☐ Flow cytometry
☒	☐ MRI-based neuroimaging

Antibodies

Antibodies used	1. Monoclonal anti-FLAG M2 monoclonal antibody, Sigma F1804, several lots were used for ChIP-seq analysis (GLI3-3xF, SMAD4-3XF)<br>2. Anti-histone H3 (acetyl K27) antibody, ChIP Grade Abcam ab4729, lot GR313984-17<br>3. Anti-Digoxigenin-AP, Fab fragments, Roche 11093274910 was used for WISH
Validation	1. Monoclonal anti-FLAG M2 monoclonal antibody was validated by using a wild-type control sample in combination with anti-FLAG M2 monoclonal antibody for ChIP-seq to detect non-specific peaks (Osterwalder et al. 2014 10.1016/j.devcel.2014.09.018).<br>2. The anti-histone H3 (acetyl K27) antibody is validated by the manufacturer in cells using chromatin from HELA cells for ChIP-qPCR.<br>3. The Anti-Digoxigenin-AP antibody detects digoxigenin-labelled riboprobes in WISH and is therefore independent of the embryo species used. It has been used by embryologists since almost three decades; Wilkinson, 1992 doi.org/10.1017/S0016672300031402

Eukaryotic cell lines

Policy information about cell lines

Cell line source(s)	G4 mouse embryonic stem cells (George et al. 2007 10.1073/pnas.0609277104); Cell vials were obtained directly from Dr. Andras Nagy, Samuel Lunenfeld Research Institute, Mount Sinai Hospital, Toronto, Canada.
Authentication	The cells were not authenticated
Mycoplasma contamination	negative for mycoplasm
Commonly misidentified lines<br>(See ICLAC register)	No commonly misidentified cell lines were used in the study

Animals and other organisms

Policy information about studies involving animals; ARRIVE guidelines recommended for reporting animal research

Laboratory animals	1. Mouse embryos were collected from pregnant mouse females in the Swiss albino background carrying the Grem1 alleles of interest at embryonic days E9.75 - E14.5.<br>2. Rabbit embryos were collected from pregnant females (New Zealand White) at gestational days D11.5-D12.0.<br>3. Pig embryos were collected from pregnant sows (Sus scrofus) at gestational days E21-E25.<br>4. Bovine embryos were collected from pregnant cows (Bos taurus) at gestational day D34. Embryos were produced by artificial insemination of cattle of the experimental dairy herd of INRA at Domaine du Pin destined to be sacrificed for meat production (mostly crosses between Holstein and Normande breeds).<br>5. Chicken embryos were collected from eggs (Gallus gallus) after incubation at Hamburger-Hamilton stages HH22-HH25.<br>6. Lizard embryos were collected for eggs (Anolis sagrei) after incubation at embryonic stages 4-6.<br>7. Python embryos were collected from eggs (Python regius) after incubation at embryonic stages 1-4.<br>8. Embryos of the brown-banded bamboo shark were collected at stages 29 and 30.<br>In all cases embryos of both sexes were used for analysis.
Wild animals	No wild animals were used in the study
Field-collected samples	No field-collected samples were used in the study
Ethics oversight	All animal experiments were performed in accordance with national laws and approved by the national/local regulatory and ethic committees/authorities. Switzerland: Regional Commission on Animal Experimentation and the Cantonal Veterinary Office of the city of Basel. Germany, rabbit embryos: German Animal Welfare Act (Tierschutzgesetz) and LAVES (Niedersächsisches Landesamt für Verbraucherschutz, Oldenburg, Germany); pig embryos: Regierung von Oberbayern - Sachgebiet 55.2 - Rechtsfragen Gesundheit, Verbraucherschutz und Pharmazie. France, bovine embryos: Comité Rennais d' Ethique en matière d'Expérimentation Animale. USA, lizard and python embryos: University of Florida Institutional Animal Care and Use Committee (IACUC); mouse embryos (Gli3 ChIP-seq): The Jackson Laboratory institutional animal care and use committee (IACUC). Japan: Experiments with bamboo shark embryos were conducted in accordance with guidelines approved by the Institutional Animal Care and Use Committee at the RIKEN Kobe Branch). All experiments were performed by members of staff trained and licensed to perform animal studies. The 3R principles were strictly implemented in all animal study design and execution. Highest animal welfare standards were implemented for animal husbandry and animal health. The group of the senior authors also implements the principles of the Basel declaration into their animal research.

Note that full information on the approval of the study protocol must also be provided in the manuscript.

ChIP-seq

Data deposition

☒ Confirm that both raw and final processed data have been deposited in a public database such as GEO.

☒ Confirm that you have deposited or provided access to graph files (e.g. BED files) for the called peaks.

Data access links<br>*May remain private before publication.*	GSE151488:<br>https://www.ncbi.nlm.nih.gov/geo/query/acc.cgi?acc=GSE151488<br>GSE151647<br>https://www.ncbi.nlm.nih.gov/geo/query/acc.cgi?acc=GSE151647
Files in database submission	The list of files submitted are as below:<br><br>ChIP-seq files in database submission with accession GSE151488<br><br>Raw files<br>CHIP_H3K27Ac_FL_HP_E11.5_rep1_R1.fastq.gz<br>CHIP_H3K27Ac_FL_HP_E11.5_rep1_R2.fastq.gz<br>CHIP_H3K27Ac_FL_HP_E11.5_rep2_R1.fastq.gz<br>CHIP_H3K27Ac_FL_HP_E11.5_rep2_R2.fastq.gz<br>CHIP_H3K27Ac_FL_HP_E11.5_Input_R1.fastq.gz<br>CHIP_H3K27Ac_FL_HP_E11.5_Input_R2.fastq.gz<br>CHIP_H3K27Ac_FL_E10.5_mm10_rep1_RS_R1.fastq.gz<br>CHIP_H3K27Ac_FL_E10.5_mm10_rep1_RS_R2.fastq.gz<br>CHIP_H3K27Ac_FL_E10.5_mm10_rep2_RS_R1.fastq.gz<br>CHIP_H3K27Ac_FL_E10.5_mm10_rep2_RS_R2.fastq.gz<br>CHIP_H3K27Ac_FL_E10.5_mm10_input_RS_R1.fastq.gz<br>CHIP_H3K27Ac_FL_E10.5_mm10_input_RS_R2.fastq.gz<br><br>Processed files<br>CHIP_H3K27Ac_FL_HP_E11.5_rep1.bigWig<br>CHIP_H3K27Ac_FL_HP_E11.5_rep2.bigWig

CHIP_H3K27Ac_FL_HP_E11.5_Input.bigWig
CHIP_H3K27Ac_FL_E10.5_rep1.bigWig
CHIP_H3K27Ac_FL_E10.5_rep2.bigWig
CHIP_H3K27Ac_FL_E10.5_control.bigWig

ChIP-seq files in database submission with accession GSE151647
Raw files

Smad4_WE_R1.fastq.gz
Smad4_WE_R2.fastq.gz
input_WE_R1.fastq.gz
input_WE_R2.fastq.gz
E11.5_LIMB_CHIP_GLI3_REP1.fastq.gz
E11.5_LIMB_CHIP_GLI3_REP2.fastq.gz
E11.5_LIMB_CTRL_INPUT.fastq.gz
E11.5_LIMB_CTRL_MOCK.fastq.gz
E10.5_LIMB_CHIP_GLI3_REP1.fastq.gz
E10.5_LIMB_CHIP_GLI3_REP2.fastq.gz
E10.5_LIMB_CTRL_INPUT.fastq.gz
E10.5_LIMB_CTRL_MOCK.fastq.gz

Processed files
GSM4587515_Smad4_WE_ChIP.bigWig
GSM4587516_Smad4_WE_input.bigWig
GSM4587517_Gli3_ChIP_E11.5_rep1.wig.gz
GSM4587518_Gli3_ChIP_E11.5_rep2.wig.gz
GSM4587519_Gli3_input_E11.5.wig.gz
GSM4587520_Gli3_mock_E11.5.wig.gz
GSM4587521_Gli3_ChIP_E10.5_rep1.wig.gz
GSM4587522_Gli3_ChIP_E10.5_rep2.wig.gz
GSM4587523_Gli3_input_E10.5.wig.gz
GSM4587524_Gli3_mock_E10.5.wig.gz

Genome browser session (e.g. UCSC)

no longer applicable

Methodology

Replicates

For all ChIP-seq analysis, high quality sequencing data was generated for n≥2 replicates

Sequencing depth

H3K27ac ChIP-seq: a total of >45 mio high quality paired-end reads of 41bp per sample was subjected to quality check and alignment using the qQCReport function of the QuasR v3.4.2 R package (Bioconductor v3.6)
SMAD4 ChIP-seq: a total of >13 mio high quality paired-end reads of 41bp per sample was subjected to quality check using FastQC v0.11.4, and Trim_Galore v0.4.1. After quality check, high quality reads (>99%) were aligned using Bowtie v2.2.9, yielding >67% of uniquely aligned reads in both ChIP and Input samples.
GLI3 ChIP-seq: a total of >20 mio high quality 76 bp single-end reads were generated for each sample. After aligning reads using BWA, >70% of the reads were obtained as uniquely aligned for each sample, with the exception of E10.5_LIMB_GLI3_REP2 (13.95% uniquely aligned reads) and E10.5_LIMB_MOCK (49.378% uniquely aligned reads).

Antibodies

1. Monoclonal anti-FLAG M2 monoclonal antibody, Sigma F1804, several lots were used for ChIP-seq analysis (GLI3-3xF, SMAD4-3XF)
2. Anti-histone H3 (acetyl K27) antibody, ChIP Grade Abcam ab4729, lot GR313984-17

Peak calling parameters

MACS2 (Zhang et al. 2008) was used for peak calling.

H3K27ac ChIP-seq: parameters used were '-q 0.01 -g 2652783500 --keep-dup all' during peak calling from MACS2v2.1.1.

SMAD4 ChIP-seq: peak calling was performed using MACS2 v2.1.1, following ENCODE recommendations with parameters -p --nomodel --extsize --call-summits -B -SPMR. A p-value threshold is set at 1e-2 and extsize value was obtained using “predictd” utility given by MACS2.

GLI3 ChIP-seq: Reads were mapped to mm10 using bwa aln. Peak calls were made using macs2 with the default parameters: genome-size = 1.87e+09, band width = 300, mfold = [5,50] and qvalue cut-off = 5.00e-02. Peak calls were made relative to mock and input controls.

Data quality

H3K27ac ChIP-seq: the peak were called with an FDR<0.01 as described in the methods ('-q 0.01')

SMAD4 ChIP-seq: 68.77% of the peaks were at FDR 5%. While 13.7% of the peaks were found with at least 5 fold enrichment and >99% of the peaks have an at least 2 fold enrichment.

GLI3 ChIP-seq: Data quality was ensured by recovery of the Gli binding site as primary motif. Total number of peaks called for each dataset with 5% FDR and fold-enrichment > 5 are as follows: E10.5_LIMB_GLI3_REP1_INPUT (5348 peaks); E10.5_GLI3_LIMB_REP2_INPUT (22,181 peaks); E11.5_LIMB_GLI3_REP1_INPUT (98,520 peaks); E11.5_LIMB_GLI3_REP2_INPUT (54,950).

Software

The publicly available softwares FastQC v0.11.4, Trim_Galore v0.4.1, Bowtie v2.2.9, MACS2v 2.1.1, SAMtools v1.7, Burrows-Wheeler Alignment (BWA) for Gli3 ChIP-seq, Picard v2.9.2, QuasR bioconductor package was used for ChIP analysis.
